# Pharmacological modulation of b-adrenoceptors as a new cardioprotective strategy for therapy of myocardial dysfunction induced by ischemia and reperfusion^1^

**DOI:** 10.1590/s0102-865020190050000005

**Published:** 2019-06-03

**Authors:** Francisco Sandro Menezes-Rodrigues, Paolo Ruggero Errante, José Gustavo Padrão Tavares, Renato Ribeiro Nogueira Ferraz, Walter José Gomes, Murched Omar Taha, Carla Alessandra Scorza, Fúlvio Alexandre Scorza, Afonso Caricati-Neto

**Affiliations:** I Fellow PhD degree, Postgraduate Program in Pharmacology, Department of Neurology and Neurosurgery, Universidade Federal de São Paulo (UNIFESP), Brazil. Conception and design of the study, analysis and interpretation of data, manuscript writing.; II Fellow PhD degree, Postgraduate Program in Pharmacology, UNIFESP, Sao Paulo-SP, Brazil. Manuscript writing.; III Fellow PhD degree, Postgraduate Program in Management of Health System (PMPA-GSS), Universidade Nove de Julho (UNINOVE), Sao Paulo-SP, Brazil. Statistics analysis.; IV PhD, Head, Division of Cardiovascular Surgery, UNIFESP, Sao Paulo-SP, Brazil. Technical procedures, interpretation of data, critical revision.; V PhD, Associate Professor, Department of Surgery, UNIFESP, Sao Paulo-SP, Brazil. Technical procedures, interpretation of data.; VI PhD, Associate Professor, Department of Neurology and Neurosurgery, UNIFESP, Sao Paulo-SP, Brazil. Analysis and interpretation of data, critical revision.; VII PhD, Associate Professor, Department of Pharmacology, UNIFESP, Sao Paulo-SP, Brazil. Critical revision.

**Keywords:** Cardiovascular Diseases, Myocardial Ischemia, Reperfusion, Receptors, Adrenergic, Drug Therapy, Rats

## Abstract

**Purpose:**

To evaluate the cardioprotective response of the pharmacological modulation of β-adrenergic receptors (β-AR) in animal model of cardiac ischemia and reperfusion (CIR), in spontaneously hypertensive (SHR) and normotensive (NWR) rats.

**Methods:**

CIR was induced by the occlusion of left anterior descendent coronary artery (10 min) and reperfusion (75 min). The SHR was treated with β-AR antagonist atenolol (AT, 10 mg/kg, IV) 5 min before CIR, and NWR were treated with β-AR agonist isoproterenol (ISO, 0.5 mg/kg, IV) 5 min before CIR.

**Results:**

The treatment with AT increased the incidence of VA, AVB and LET in SHR, suggesting that spontaneous cardioprotection in hypertensive animals was abolished by blockade of β-AR. In contrast, the treatment with ISO significantly reduced the incidence of ventricular arrhythmia, atrioventricular blockade and lethality in NWR (30%, 20% and 20%, respectively), suggesting that the activation of β-AR stimulate cardioprotection in normotensive animals. Serum CK-MB were higher in SHR/CIR and NWR/CIR compared to respective SHAM group (not altered by treatment with AT or ISO).

**Conclusion:**

The pharmacological modulation of β-AR could be a new cardioprotective strategy for the therapy of myocardial dysfunctions induced by CIR related to cardiac surgery and cardiovascular diseases.

## Introduction

Cardiovascular diseases (CVD) are the major cause of death in the world in developed and developing countries, and approximately 17 million people die each year from CVD and by 2030 this figure is expected to reach 26 million^1^. Arterial hypertension constitutes an important risk factor for CVD^2^ ^,^ ^3^. Hypertension is characterized by systolic blood pressure equal to or above than 140 mm Hg and/or diastolic blood pressure levels equal to or above than 90 mmHg. 

Hypertension increases the risk of cardiac events, myocardium infarction and other ischemic cardiac diseases, which are treated by surgical interventions. These interventions involve the exposure of the heart for variable periods of cardiac ischemia and reperfusion (CIR). The increase of duration and severity of CIR are demonstrate to elicit increment of irreversible myocardial damage, and susceptibility to further injury during reperfusion, severely compromising cardiac structure and function^4^ ^,^ ^5^. Hypertension is characterized by autonomic dysfunctions, especially the sympathetic hyperactivity and involves tachycardia, increased peripheral vascular resistance, and elevated plasma catecholamine concentrations^6^ ^,^ ^7^. Interestingly, some studies have suggested that sympathetic hyperactivity can be involved in cardioprotective response^8^ ^-^ ^10^. 

Following this line of reasoning, *in vitro* and *in vivo* studies using animal models of CIR showed that treatment with β-adrenoceptor (β-AR) agonist isoproterenol (ISO) or ischemic preconditioning (IPC) protocol reduced infarct size, cardiac necrosis and ventricular arrhythmia (VA), reinforcing the concept of sympathetic mechanisms involvement in cardioprotection^8^ ^-^ ^11^. In this sense, to investigate the role of β-AR in cardioprotection, we studied the effects of agonists and antagonist of β-AR on the cardiac arrhythmias, lethality (LET) and serum levels of cardiac injury markers in spontaneously hypertensive (SHR) and normotensive (NWR) rats submitted to CIR.

## Methods

All experimental protocols were approved by Ethical Committee of the UNIFESP (0065/12). The study enrolled adult male NWR and SHR weighting between 307 to 351 g with 12 to 14-week-old obtained from Centro de Desenvolvimento de Modelos Animais para Medicina e Biologia (CEDEME) of the Universidade Federal de São Paulo (UNIFESP). These animals were maintained under standard conditions of nutrition, hydration, temperature, light and humidity, and in accordance to normalization approved were. 

The NWR and SHR were randomized into 8 groups: 1) NWR/SHAM (n=12): NWR submitted to surgical procedures, but not underwent CIR; 2) NWR/CIR (n=17): NWR submitted to surgical procedure for CIR induction; 3) NWR/CIR+ISO (n=12): NWR submitted to surgical procedure for CIR induction and treated with β-AR agonist isoproterenol (ISO, 0.5 mg/kg administrated intravenously 5min before of CIR); 4) NWR/CIR+AT+ISO (n=12): NWR submitted to surgical procedure for CIR induction and treated with β-AR antagonist atenolol (AT, 10 mg/kg administrated intravenously 10 min before of CIR) and ISO (0.5 mg/kg administrated intravenously 5 min before of CIR); 5) SHR/SHAM (n=12): SHR submitted to surgical procedures, but not underwent CIR; 6) SHR/CIR (n=24): SHR submitted to surgical procedure for CIR induction; 7) SHR/CIR+AT (n=12): SHR submitted to surgical procedure for CIR induction and treated with AT (10 mg/kg administrated intravenously 5 min before of CIR); 8) SHR/CIR+ISO (n=6): SHR submitted to surgical procedure for CIR induction and treated with ISO (0.5 mg/kg administrated intravenously 5min before CIR).

### Non-invasive method of measuring blood pressure

 Systolic blood pressure (SBP) in non-anesthetized both NWR and SHR was measured by non-invasive tail-cuff plethysmography using the “Mouse and Rat Tail Cuff Method Blood Pressure System” (System IITC Inc., California, USA), in accordance with method described by Musial et al ^12^.

### Method for induction of CIR

 The method for induction of CIR has previously been described by our lab^11^ ^,^ ^13^ ^-^ ^15^. Rats were anesthetized with urethane (1.25 g/kg), and fixed in the supine position. After intubation (Jelco 14G, USA), rats were mechanically ventilated using a mechanic ventilator Insight model EFF 312 (Insight Equipamentos Científicos, Ribeirao Preto-SP, Brazil). After stabilization for 15 min, thoracotomy was performed to place the vascular tourniquet (4/0 braided silk suture attached to a 10-mm micropoint reverse-cutting needle, Ethicon K-890H, USA) around the left anterior descending coronary artery to induce ischemia. After of 10 min of cardiac ischemia, the tourniquet was removed to allow coronary recirculation for 75 min (cardiac reperfusion). The cardiac electrical activity in all groups studied was monitored by electrocardiogram (ECG) system using a method previously described by our lab^11^ ^,^ ^13^ ^-^ ^15^. ECG analysis was performed during 100 min of duration (stabilization for 15 min, cardiac ischemia for 10 min and cardiac reperfusion for 75 min). The ECG was recorded using a biopotential amplifier by means of needle electrodes placed subcutaneously on the limbs. Successful surgical obstruction of the coronary artery was validated by ECG alterations (increase in R wave and ST segment) caused by cardiac ischemia. The body temperature was maintained at 37.5ºC with a heated operating platform and appropriate heating lamps and was evaluated routinely via a rectal thermometer.

### Evaluation of cardiac function by ECG analysis

 The ECG data were recorded using an acquisition system AqDados 7.02 (Lynx Tecnologia Ltda., Brazil), an acquisition system AqDados 7.02 (Lynx Tecnologia Ltda., Brazil), and analyzed using the software AqDAnalysis 7 (Lynx Tecnologia Ltda., Brazil). Using this software, the heart rates were evaluated, as well as incidence of ventricular arrhythmias (VA), atrioventricular block (AVB) and lethality (LET), in response to CIR. The ventricular fibrillation, *torsades de pointes*, and ventricular tachycardia parameters were considered only as VA. 

### Evaluation of cardiac lesion biomarkers

 After ECG recording, blood sample were collected from the abdominal aorta artery, and centrifuged (2.500 rpm, for 40 min, at 5°C) for serum isolation. The serum was stored at -20°C for biochemical analysis. Quantitative determination of creatine kinase MB fraction (CK-MB) was performed using a kinetic-UV method, measured at 340 nm (Kit Katal Biotecnológica Ind. Com. Ltda., Belo Horizonte-MG, Brazil).

### Statistical analysis

 The incidence of VA, AVB, and LET were statistically evaluated using the Fisher’s exact test (p<0.05). Ratio between heart mass and body mass (HM/BM) was statistically evaluated using Student’s test (p <0.05), and serum concentration of CK-MB are expressed as mean ± standard error of mean (SEM), and analysis of variance (ANOVA) was applied, followed by the Tukey post-test (p <0.05) using Prism 5.0 software (GraphPad, USA).

## Results

### Blood pressure and cardiac mass in SHR and NWR

**Figure 1:**
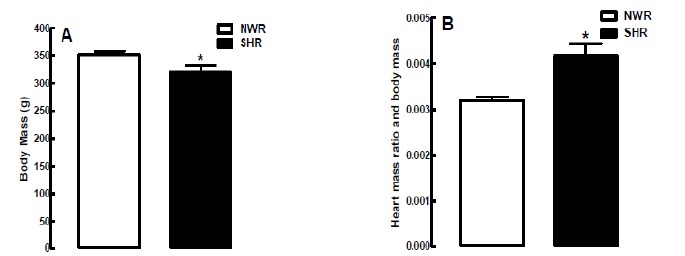
Histogram showing the values of body mass (**A**) and heart mass ratio and body mass (HM/BM) (**B**) in NWR and SHR. The results are expressed as mean ± standard error of the mean, and Student’s test was applied to evaluate the **s**tatistically different from the NWR,*p <0.05 (n = 40 samples for each group).

 The systolic blood pressure (SBP) value was 199 ± 4 mm Hg in SHR and 132 ± 2 mm Hg in NWR. Cardiac hypertrophy represents another phenotypic characteristic of hypertension. In this work, the cardiac mass was 1,335 ± 110 mg in SHR and 1,121 ± 98 mg in NWR, showing that cardiac mass in SHR was 19% greater compared to NWR (figure 1). Although the body mass value was 11% lower in SHR (320 ± 13 g) compared to NWR (351 ± 7 g), the ratio between heart mass and body mass (HM/BM) was 31% higher in SHR (0.0041) compared to NWR (0.0031). These phenotypic characteristics confirm the hypertensive state in SHR (Fig. 1).

### Cardiac function in SHR and NWR

**Figure 2:**
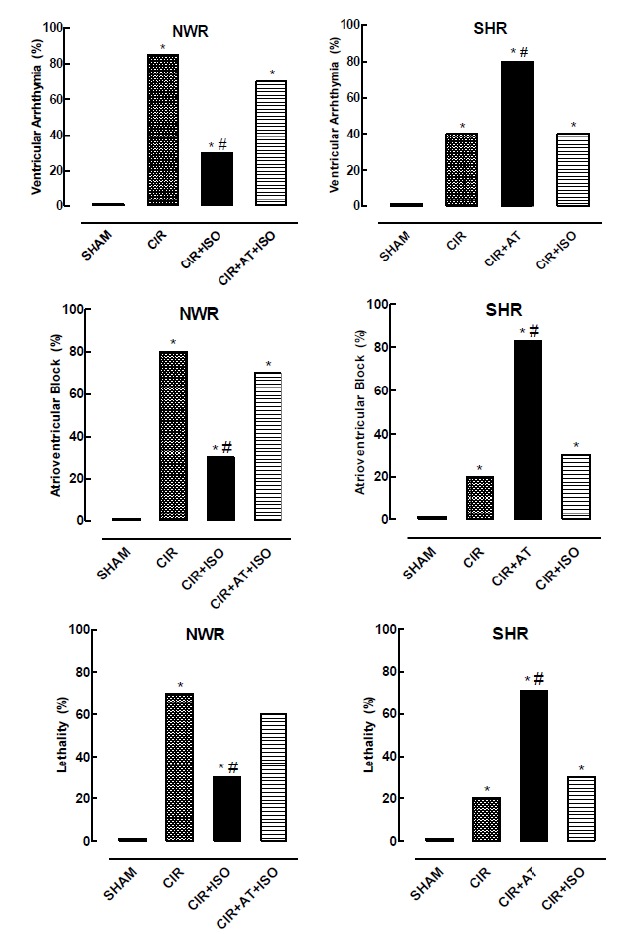
Incidence of ventricular arrhythmias (AV), atrioventricular block, and lethality in the NWR/SHAM, NWR/CIR, NWR/CIR+ISO, NWR/CIR+AT+ISO, SHR/SHAM, SHR/CIR, SHR/CIR+AT, NWR/CIR+ISO groups. The results are expressed as mean, and Fisher’s exact test was applied. * Statistically different from the NWR/SHAM or NWR/CIR and SHR/SHAM or SHR/CIR, p <0.05. # Statistically different from the CIR, p <0.05. (n= 12 to 24 for each group).

 ECG analysis showed that the incidence of VA, AVB and LET was 0% in SHR/SHAM and NWR/SHAM groups (Fig. 2). The incidence of VA, AVB and LET in SHR/CIR group was 40%, 20% and 20%, respectively, and in NWR/CIR group (85%, 80% and 70%, respectively (Fig. 2). These results show that the incidence of VA, AVB and LET significantly lower in SHR compared to NWR, suggesting the presence of spontaneous adaptive cardioprotective response in hypertensive. To test this hypothesis, we studied the effects of drugs that interfere on the activity of cardiac β-AR in SHR and NWR. It is well known that hypertension in SHR is related to sympathetic hyperactivity^16^ ^-^ ^18^. Then, we treated the SHR with AT before CIR to reduce this sympathetic hyperactivity due to blockade of the β-AR in cardiovascular tissues. We observed that the AT significantly increased the incidence of VA (from 40% to 80%), AVB (from 20% to 83%), and LET (from 20% to 71%) in SHR/CIR group (Fig. 2). These results indicate that pharmacological blockade of β-AR abolished the spontaneous adaptive cardioprotective response in hypertensive animals, indicating the direct involvement of the β-AR in this response. 

To confirm the involvement of the β-AR in sympathetic mechanisms involved in the cardioprotective response, we treated the NWR with ISO before CIR to increase the sympathetic activity, simulating the sympathetic hyperactivity in SHR. We observed that this treatment significantly reduced the incidence of VA (from 85% to 30%), AVB (from 80% to 30%), and LET (from 70% to 30%) in NWR/CIR group (Fig. 2), indicating that the stimulation of the cardiac β-AR in normotensive animals induces cardioprotective response. To confirm this hypothesis, the NWR/CIR group was treated with AT before of the stimulation of β-AR by ISO. These assays showed that the pretreatment with AT abolished the reduction in the incidence of VA, AVB and LET induced by ISO in NWR/CIR group (Fig. 2), indicating that the blockade of cardiac β-AR abolished the cardioprotective response induced by pharmacological stimulation of these receptors in normotensive animals. The results obtained in NWR and SHR submitted to CIR confirm the involvement of the cardiac β-AR in the cardioprotective response (Fig. 2).

### Cardiac lesion biomarker in SHR and NWR

**Figure 3:**
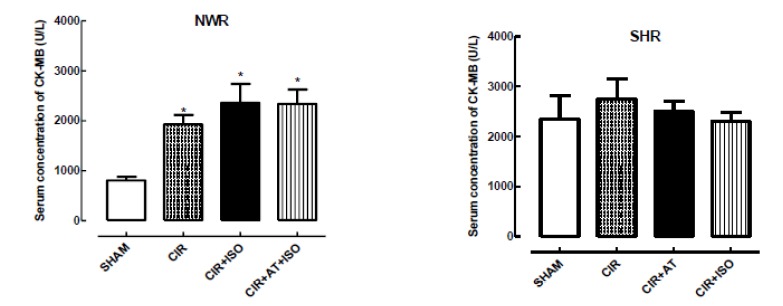
Serum concentration of CK-MB in the NWR/SHAM, NWR/CIR, NWR/CIR+ISO, NWR/CIR+AT+ISO, SHR/SHAM, SHR/CIR, SHR/CIR+AT, NWR/CIR+ISO groups. The results are expressed as mean ± standard error of the mean, and analysis of variance (ANOVA) was applied, followed by the Tukey post-test. * Statistically different from the NWR/SHAM or NWR/CIR and SHR/SHAM or SHR/CIR, p <0.05. ^#^ Statistically different from the CIR, p <0.05. (n= 6 to 8 samples for each group).

 The biochemical evaluation of cardiac injury biomarker CK-MB showed important differences between SHR and NWR in response to CIR and the treatment with AT or ISO. Serum concentration of CK-MB were significantly higher in the NWR/CIR compared to NWR/SHAM group (Fig. 3), indicating that CIR produced an increase in the serum concentration of CK-MB in normotensive animals. In contrast, serum concentration of CK-MB was similar in the SHR/SHAM and SHR/CIR (Fig. 3), indicating that CIR was not able to increase the serum concentration of CK-MB in hypertensive animals. Comparisons of serum concentration of CK-MB between SHR/SHAM and NWR/SHAM groups, showed that CK-MB is increased in hypertensive animals independently of CIR (Fig. 3), suggesting that hypertension could stimulate the increase in the serum concentration of CK-MB. However, the involvement of sympathetic mechanisms in this response is unclear. 

To test the possible involvement of sympathetic mechanisms in the serum levels of CK-MB, we studied the effects of AT and ISO on the serum concentration of this cardiac injury biomarker in NWR and SHR submitted to CIR. We observed that the pretreatment with AT not altered serum concentration of CK-MB in SHR/CIR group (Fig. 3), indicating that the β-AR does not interfered in the synthesis of CK-MB in hypertensive animals. We also studied the effects of the treatment with ISO on the serum levels of CK-MB in NWR submitted to CIR. This treatment does not alter serum levels of CK-MB compared to NWR/CIR (Fig. 3), indicating that the β-AR does not interfered in the serum concentration of CK-MB in normotensive animals. 

## Discussion

Using *in vivo* animal model of CIR, we investigated in the present study the involvement of cardiac β-AR in the cardioprotective response in normotensive (NWR) and hypertensive (SHR) animals. This study showed that the CIR increases the incidence of VA, AVB and LET in NWR and SHR, but the incidences of all these parameters were significantly lower in SHR. These findings suggest, for the first time, the presence of a spontaneous adaptive cardioprotective response in hypertensive animals. The SHR strain is the main study model of primary hypertension in humans^19^. In this model, the hypertension is related to changes in cardiovascular parameters, determinants of systemic arterial pressure, with increased total peripheral resistance with normal or decreased cardiac output^20^. The elevation of blood pressure levels in SHR begins approximately in the 5th or 6th week of life, presenting high pressure levels between the 7th to the 15th weeks and plateau around the 20th to the 28th week, at which age, the blood pressure values reach their maximum levels. Due to the onset of hypertension, the SHR model develops progressive cardiac hypertrophy that progresses to heart failure^21^ ^,^ ^22^. Similarly to human hypertension, the SHR used in present work presented SBP values above 140 mm Hg and cardiac hypertrophy, confirming the hypertensive state in SHR^19^. 

Because of the hypertension, the SHR develops cardiac hypertrophy that progresses to heart failure^21^ ^,^ ^22^. However, the main triggering factor of hypertensive conditions in the SHR is the increase in peripheral vascular resistance due to sympathetic hyperactivity, one of the main features of these animals^23^. Therefore, SHR presents a significant reduction in microvascular density and blood supply in several organs, like skeletal muscle^24^, gut^25^, skin^26^ and heart^27^. The reduced density of myocardial capillaries leads to the inability of microcirculatory growth, which does not accompany the cardiac hypertrophy triggered by the hypertension^28^. 

It is well established that the hyperactivity sympathetic is involved in the pathogenesis of hypertension in humans and animal hypertension models, such as SHR^16^ ^-^ ^18^. For example, the sympathetic activity and discharge from cervical, splanchnic and renal sympathetic neurons is significantly increased in SHR^16^ ^-^ ^18^. This sympathetic hyperactivity is also involved in other cardiovascular dysfunctions, including the acute myocardial infarction (AMI). However, a growing number of evidences suggest that the sympathetic hyperactivity associated to cardiovascular diseases could stimulate the cardioprotective response^8^ ^-^ ^11^ ^,^ ^29^. However, the molecular mechanisms involved in this cardioprotective response remains unclear. 

To investigate the molecular mechanisms involved in this cardioprotective response, we studied the effects of agonist (ISO) and antagonist (AT) of β-AR on the incidence of VA, AVB and LET in SHR submitted to CIR. The treatment with β_1_-AR antagonist AT increased the incidence of VA, AVB and LET in SHR submitted to CIR, confirming that the blockade of cardiac β_1_-AR abolished the spontaneous cardioprotection in hypertensive animals. The treatment with β_1_/β_2_-AR agonist ISO does not change the incidence of VA, AVB and LET in SHR submitted to CIR, probably due to preexistent spontaneous cardioprotective response in hypertensive animals. These results suggest that sympathetic hyperactivity associated to primary hypertension could induce a set of adaptive responses able to attenuate myocardial dysfunctions and lesions caused by CIR, including an increased density/function of cardiac β-AR.

It is important to mention that prior exposure to brief CIR periods conferred spontaneous cardioprotection against cardiac lesions produced by prolonged CIR^8^. This procedure called ischemic preconditioning (IPC) has become one of the experimental non-pharmacological strategies to reduce the area of injury produced by CIR. In addition, it has been shown that IPC can confer cardioprotection by decreasing the incidence of arrhythmias caused by CIR^11^ ^,^ ^30^ ^,^ ^31^. These events are characteristic of IPC that is able to provide cardioprotection, the cardioprotective effects cause by was observed in several types of cardiac preparations (culture of cardiomyocytes and isolated hearts) and animal species, among which are the rats^11^ ^,^ ^32^ ^-^ ^34^. Data from the literature have shown that blocking β_1_-AR decreases cardioprotection promoted by IPC, one of which such blocking promotes negative effects on cardiac tolerance to IPC, since this blockade can exert negative effects on functional CIR tolerance and signaling pathways involved in the cardioprotection caused by activation of β_1_-AR and IPC^35^ ^-^ ^39^.

*In vitro* studies demonstrated that prior exposure for 1 to 2 short cycles of ischemia (5 min) and reperfusion (10 min) reduced the incidence of ventricular fibrillation from 42% to 17% with 2 cycles and completely abolished this type of arrhythmia with 1 cycle in isolated rabbit hearts submitted to ischemia for 30 min followed by reperfusion for 45 min^32^. In addition, it was showed that the pre-exposure of hearts isolated from Wistar rats to 1 cycle of ischemia (5 min) and reperfusion (5 min) reduced the number of premature ventricular beats (518 ± 71 bpm for 195 ± 40 bpm) and reduced the incidence (from 100% to 25%) and duration (from 44 ± 9 s to 18 ± 5 s) of ventricular tachycardia in isolated hearts submitted to 30 min of ischemia^40^. The cardioprotective efficacy of IPC by prior exposure to 2 cycles of ischemia (5 min) and reperfusion (5 min) in Wistar rats submitted to cardiac ischemia for 30 min and reperfusion for 24 h significantly reduced incidence of AV and LET (from 50% to 12%), as well as the area of the myocardial injury (from 40% to 25%)^34^. 

To confirm the involvement of β-AR in cardioprotective response, we also studied the effects of ISO and AT on the incidence of VA, AVB and LET in NWR submitted to CIR. The treatment with ISO significantly reduced the incidence of VA, AVB and LET in NWR submitted to CIR, suggesting that the pharmacological activation of β-AR stimulated the cardioprotective response in normotensive animals. This effect of ISO was abolished by the pretreatment with AT, confirming that the blockade of cardiac β-AR abolished the pharmacological cardioprotective response in NWR. 

Several experimental studies have suggested the involvement of different subtypes of β-AR in the cardioprotective response^29^ ^,^ ^41^. It was showed that the treatment with the β_2_-AR agonist clenbuterol produced a significant cardioprotective response against CIR injury by the β_2_-AR-Gi-protein signaling stimulation in rats^29^. The transient activation of β_1_/β_2_-AR in hearts preconditioned for 5 min with agonists of β_1_/β_2_-AR (ISO), β_1_-AR (denopamine), β_2_-AR (formoterol) elicited cardioprotective response against CIR injury in rats^41^. This study also suggested the involvement of nitric oxide (NO) in β_1_/β_2_-AR ischemic preconditioning (IPC) and the Gi protein in β_2_-AR mediated preconditioning in rats^41^. Salie *et al*.^41^ suggested that the intracellular messengers coupled to β-AR such as protein kinase A (PKA) and PI3-K is essential for cardioprotective response mediated by cardiac β_1_/β_2_-AR. 

The selective stimulation of β_3_-AR is able to reduce infarct size in animal model of CIR. The pretreatment with the β_3_-AR agonist BRL37344 (5 μg/kg) reduce infarct size in wild-type mice with left ventricular function improved in β_3_-AR agonist treated mice. The incubation with β_3_-AR agonist significantly reduces cell death in isolated adult mouse cardiomyocytes during hypoxia-reoxygenation periods and decreased susceptibility to deleterious opening of the mitochondrial permeability transition pore, via a mechanism dependent on the Akt-NO signaling pathway. The administration of BRL37344 decrease infarct size and improved long-term left ventricular contractile function^42^. 

In addition, we observed that the serum concentration of CK-MB was significantly higher in the NWR submitted to CIR compared to its respective SHAM, suggesting that CIR induced cardiac injury. In contrast, serum concentration of CK-MB was similar in the SHR submitted to CIR and SHR/SHAM. Comparisons of serum concentration of CK-MB between SHR submitted to CIR and NWR/SHAM showed that the serum concentration of CK-MB is increased in hypertensive independently of CIR, suggesting that hypertension could stimulate the increase of CK-MB. These results agree with the results showed in the literature in high performance athletes, which according to our thesis these athletes present the IPC-induced cardioprotection similarly to those observed in hypertensives^43^ ^-^ ^45^. To test the possible involvement of sympathetic mechanisms in the serum levels of cardiac injury biomarker, we studied the effects of AT or ISO on the serum concentration of CK-MB. To reduce sympathetic hyperactivity in SHR, the cardiac β-AR was blocked by AT administration before CIR. In the SHR, the treatment with AT before CIR does not alter serum levels of CK-MB. These results suggest that blockade of β-AR does not interfere in the synthesis of cardiac injury markers in hypertensive animals. The treatment with ISO to increase the sympathetic activity does not alter serum levels of CK-MB in NWR submitted to CIR. Thus, these findings could lead to therapeutic strategies to prevent both cardiac arrhythmias and mortality in AMI in hypertensive patients. In addition, this strategy could be used in cardiac surgery to prevent the myocardial dysfunction induced by CIR.

## Conclusion

These results support the concept that β-AR are involved in cardioprotective response. Therefore, pharmacological modulation of these receptors could be a new cardioprotective strategy for the therapy of myocardial dysfunction induced by CIR related to cardiac surgery and cardiovascular diseases in humans, such as ischemic cardiac diseases and primary hypertension. 
